# Self-positioning in the context of double duty caregiving—Secondary analysis of a scoping review

**DOI:** 10.1007/s00391-025-02441-5

**Published:** 2025-04-30

**Authors:** Nicole Ruppert, Martina Roes

**Affiliations:** 1 Fakultät für Gesundheit, Department für Pflegewissenschaft, Universität Witten-Herdecke, Alfred-Herrhausen-Straße 50, 58455 Witten, Germany; 2 Arbeitsgruppe Implementierungswissenschaft & Personzentrierte Demenzversorgung, Deutsches Zentrum für Neurogenerative Erkrankungen e. V. (DZNE), Witten, Germany

**Keywords:** Professional nurse, Family caregiver, Identity, Dual role, Characteristics, Professionell Pflegende, Pflegende Angehörige, Identität, Doppelrolle, Charakteristika

## Abstract

**Background:**

The needs and support requirements of double duty caregivers (DDC) by healthcare professionals differ depending on whether they identify themselves as a professional nurse or family caregiver in private care arrangements. Questions of identity are usually not addressed by the DDCs. Resulting misunderstandings have the potential for conflict, which places additional strain on DDCs.

**Aim:**

The aim of this article is to present the essential characteristics of DDCs based on a model corresponding to their identity either as a professional nurse or family caregiver in a private care arrangement.

**Methods:**

A secondary analysis of 32 articles identified after a systematic literature search for a scoping review on the topic was carried out. A total of 18 articles were included in the qualitative content analysis according to Kuckartz and Rädiker.

**Results:**

A model of * Continuum of Self-Positioning of Double Duty Caregivers* was developed. It describes the characteristics DDCs show in four contexts: caregiving context, family context, healthcare system context, own workplace. Depending on the understanding of their own role in the different care settings, they are able to develop a better understanding of their identity either as a professional nurse or family caregiver, whereby the transitions are fluid.

**Conclusion:**

There are characteristics that can be assigned to the respective presumed identity of the DDCs. Knowing this enables healthcare professionals and family members to interact appropriately with DDCs and thus minimize the potential for conflict.

## Abstract (de)

**Hintergrund:**

Die Bedürfnisse von Double Duty Caregivers (DDC) und dementsprechend die Unterstützungsbedarfe durch Fachpersonen im Gesundheitswesen unterscheiden sich, je nachdem, ob sie sich als Pflegefachperson oder pflegende Angehörige im privaten Pflegearrangement identifizieren. Häufig wird dies von den DDCs jedoch nicht thematisiert. Daraus resultierende Missverständnisse in der Interaktion haben Konfliktpotenzial, welches DDCs zusätzlich belastet.

**Ziel:**

Ziel dieses Beitrags ist es, anhand eines Modells grundlegende Charakteristika von DDCs darzustellen, die Fachpersonen im Gesundheitswesen dabei unterstützt zu erkennen, ob sich DDCs eher als Pflegefachpersonen oder pflegende Angehörige im privaten Pflegearrangement identifizieren.

**Methoden:**

Sekundäranalyse von 32 Artikeln, die nach einer systematischen Literaturrecherche für ein Scoping Review zum Thema identifiziert wurden. Insgesamt 18 Artikel wurden in die qualitative Inhaltsanalyse nach Kuckartz und Rädiker eingeschlossen.

**Ergebnisse:**

Es wurde ein Modell des *Kontinuums der Selbstpositionierung von Double Duty Caregivers *entwickelt. Das Modell beschreibt Charakteristika von DDCs, die vier Bereichen *(Pflegekontext, Familienkontext, Gesundheitssystem, eigener Arbeitsplatz)* zugeordnet werden können. Je nach Verständnis der eigenen Rolle als DDC, entwickeln sie eine Identität als Pflegefachperson oder pflegende Angehörige, wobei die Übergänge fließend sind.

**Schlussfolgerung:**

Es gibt Charakteristika, die Hinweise auf die mutmaßliche Identität der DDCs geben können. Das Wissen darüber ermöglicht Gesundheitsfachpersonen mit DDCs adäquat zu interagieren und so Konfliktpotenziale zu minimieren.

## Introduction

There are increasing numbers of nurses who care for relatives in need of care in addition to their professional nursing duties. They are called double duty caregivers (DDCs). Nurses who are DDCs, are challenged by these two roles, as a family caregiver and a professional nurse, which can be very burdensome and exhausting [7, 10, 13, 20, 26]. As a consequence some DDCs decide to reduce their workload as a nurse or quit the job altogether [2]. This, in turn, increases the staff shortage in the healthcare system, which recently led to a growing interest of employers and politicians to support DDCs with their double duty responsibilities [2, 21, 22].

Since the early 1990s studies and reviews have been conducted to record and evaluate the experiences, specific challenges, demands and needs of DDCs to recommend appropriate support measures and interventions (such as family care leave) to initiate the implementation [3, 5, 7]. A general finding of most studies is that both roles, as a family caregiver and a nurse, are inseparably entwined [8–10, 13, 14, 16, 18–20]; however, there are some factors (e.g., personality, personal wishes and preferences or the relationship with the family member in need of care) as well as the context of the general care situation (e.g., living together in the same household) that influences how DDCs experience their roles [9, 13, 14, 16, 26]. Furthermore, DDCs seem to preferably focus on one of the two roles due to the expectations of all involved, their own moral concepts (e.g., sense of duty), and their own understanding of caring [9, 10, 13, 14, 19, 20, 26]. Depending on the assumed role, which is not always self-chosen, the needs, demands of the support provided and expectations of interaction with healthcare personnel differ [5, 7–10, 12, 17–20]. A key result of a recently published scoping review [18] is that DDCs complain that they do not receive enough support to cope with their special challenges [5, 7–10, 17, 19, 20]. This is correlated, among other things, with gender and is associated with social norms [23, 26]. Beyond that, they experience that their personal needs are not recognized, respected or responded to by healthcare workers [7, 9, 10, 14, 17–19]. Furthermore, it seems that DDCs often do not voice their needs [1, 8, 9, 12, 18, 20], mostly because they find it difficult to ask for help [5], are afraid of being perceived as overburdened or incapable of expressing their needs [4] or are guided by norms and expectations [7, 26] that sometimes do not correspond to their own wishes. For some DDCs, this goes hand in hand with the permanent question of whether or not to reveal themselves as a professional nurse [4, 9, 12, 18, 20]. As a consequence, healthcare workers do not know or can only guess what role DDCs currently prefer or have assumed and even if they were aware of useful measures, it would be unclear which would be the most appropriate in each situation [18]. This dilemma hinders them from acting accordingly, which can cause miscommunication and conflicts. Therefore, the main question of this secondary review analysis was the following: what characterizes DDCs who identify themselves primarily as a) family caregivers or b) nurses in their private care arrangements?

The aim of this article is to present specific characteristics of DDCs, depending on whether they identify as a professional nurse or a family caregiver in the private care arrangement, based on a literature search. The results are intended to provide healthcare professionals with information on how to recognize the presumed positioning of DDCs and react accordingly. Predictors and consequences of dual roles will not be further discussed in this article, since these can be found in our scoping review 18.

## Methods

**Fig. 1:**
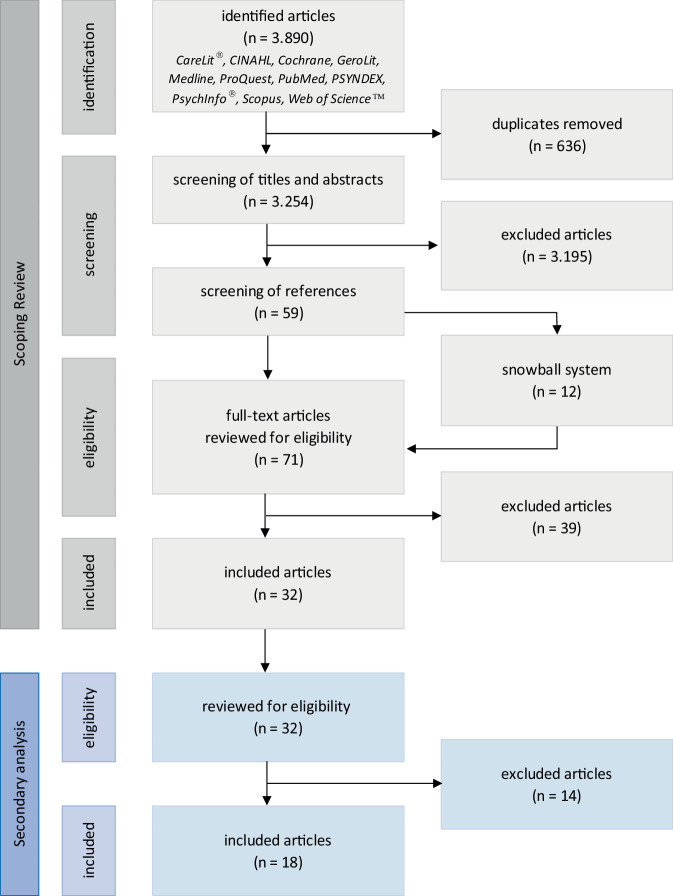
PRISMA flowchart of previous scoping review and secondary analysis

The basis for answering the research question of this secondary review analysis was a reprocessing of the results of 32 articles included in the previously published scoping review [18]. A complete reference list of the articles included in the scoping review as well as a tabular summary of all articles in terms of research designs, methods used, samples and relevant results are available online in the supplementary information to the article 10.1007/s00391-024-02382-5. The aim of the scoping review was to provide a general understanding of the experiences and challenges faced by DDCs. A systematic literature search was carried out in 11 specialized databases according to the Joanna Briggs Institute (JBI) methodology [6] and using the snowball system (see Fig. 1). The search was limited to articles published in English and German between 1995 and 2023. Articles were included if they dealt with the experiences, challenges and own understanding of the role of professional caregivers who also care for relatives. The findings of the literature search were assigned to six complexes of topics: emotional and personal aspects, demands and expectations, personal needs, role identification, nursing expertise and interaction with professionals in the healthcare system [18]. One of the findings was that healthcare workers do not realize or know that DDCs have different needs and support demands depending on how they have positioned themselves in the private care arrangement and therefore DDCs often do not feel sufficiently supported and valued [18]. For this reason, a secondary analysis was conducted with the intention of identifying and describing characteristics that provide indications as to whether the respective DDCs see themselves more as nurses or more as family caregivers in the private care arrangement. The re-evaluation of the 32 articles included in the scoping review was accordingly carried out using newly formulated inclusion and exclusion criteria [18].

Inclusion criteria: all articles that described the behavior or provided other information on the characteristics of DDCs, regardless of whether addressed by the DDCs themselves or as a conclusion of the researchers, were included.

Exclusion criteria: all articles that focused on other topics related to double duty caregiving, such as general requirements on DDC, working conditions and their adjustments as well as support services, were excluded.

In total, 18 articles [1, 3, 4, 7–10, 12–14, 16, 17, 19, 20, 23–26] could be included in the secondary review analysis (see Fig. 1). Of these articles 16 were qualitative studies, 1 was a meta-ethnography and 1 a review. In addition, 6 were from Australia, 5 from Canada, 2 from the USA and 1 each from Great Britain, New Zealand, Portugal, Sweden and Switzerland. In four cases, there were two studies each analyzing the same data sets but using different research questions ([13] and [14]; [16] and [17]; [20] and [24]; [25] and [26]). The analysis and categorization were inductively carried out using the structuring qualitative content analysis according to Kuckartz and Rädiker [15].

## Results

The included articles point out that it seems that DDCs do not see themselves only as a family caregiver or as a nurse, they always identify with both sides [10, 12–14, 16, 20, 23, 26]. This does not change, even when they lean more to one side or the other. The decision in favor of taking on one of the two roles depends on various factors: not only personality, the relationship with the family member in need of care and the general care situation have an influence on this decision making [9, 10, 13, 14, 16, 20, 23]. Personal wishes and preferences, expectations of all involved persons as well as moral concepts, a sense of duty and one’s own understanding of care also play important roles in identifying and positioning oneself as a family member or nurse in private care arrangements [1, 3, 7–10, 12–14, 16–20, 23, 25, 26]. Furthermore, it seems that the assumption of a role has an effect on the responsibilities, activities carried out and behavior shown within the care arrangement [18, 23].

The analysis of the included articles provided several different characteristics, behaviors and actions depending on whether the DDC identified more as a family member or as a nurse. The attribution of the characteristics is based on the DDCs statements of the activities and behaviors they have predominantly adopted from the perspective of their respective position as well as on the explanations and conclusions of the authors of the studies. The aspects shown for both roles can be assigned to five overarching themes: a) caregiving context, b) family context, c) healthcare system context, d) own workplace and e) fluidity of self-positioning.

### Caregiving context (a)

The DDCs, as family caregivers, see themselves in the context of care as those who primarily provide physical, nursing and medical care [17, 23, 26]. This includes, for example, personal hygiene, insulin injections and basic activities such as working around the house or accompaniment to treatment and doctor visits [14, 17, 23, 26], but they also see their duty in just being there, holding hands, etc. [17, 19]. They just want to spend as much time as possible with their relative in need of care, because the two are usually very close [7, 14, 17, 26]; however, as a nurse in a care arrangement, DDCs assume responsibility and act as coordinators and organizers [3, 14, 16, 19, 20, 23, 26]. They also act as advocates for the relative in need of care [3, 13, 14, 19, 20, 26]. After all, they are the persons who have everything in view and therefore distribute and delegate tasks [20, 23].

### Family context (b)

The DDCs who identify as family caregivers within the family context are usually in close contact with their relatives and share tasks equally [20, 23]. It is important that they work together and support other relatives [1, 20]. Also, they want to be there for everyone else, to encourage, comfort and support wherever possible [25]. Mostly, they are a family member just like the others and only share their expertise when asked [8, 20, 24]. If DDCs see themselves more as nurses, they take on tasks such as informing and counselling other family members [1, 20], coordinating and organizing in the care arrangement [1, 19, 20, 23, 26] and taking the lead in decision-making [1, 19, 20, 26], for example, regarding treatment planning or within the nursing process. In other words, they are the interface and connection between their family and healthcare workers, in both directions.

### Healthcare system context (c)

When DDCs are in contact with healthcare workers, it makes a difference whether this happens in a third-party facility or at their own workplace, as described later. The DDCs who identify themselves as family caregivers often appear insecure and gratefully accept any support and encouragement [3, 9, 13, 17, 19, 20]. They are also rather reserved and usually try to relieve the staff [8, 9, 12, 19]. In addition, they try to hide their nursing profession [9, 12, 20] but give themselves away more easily, for example, by using technical language or terms. The DDCs who assume the role of a nurse in the care arrangement are more confident. They often reveal themselves as nurses from the beginning [12]. They can also be very demanding when it comes to, for example, providing detailed information or services to their relatives [1, 3, 9, 10, 12, 13, 16, 19, 20]. Furthermore, they are not afraid to criticize or intervene practically if they believe that is necessary in the situation [3, 9, 12, 13, 16, 19, 20]. It must be taken into account that they always only have the well-being of their relative in mind.

### Own workplace (d)

As mentioned above, it is easier for DDCs to act on their own workplace, although sometimes they do not really want to talk about private matters [1, 4]. In other cases, DDCs as family caregivers seek an exchange with colleagues or other like-minded people in their institution to receive appreciation, encouragement and tips [1, 7, 8, 19]. The DDCs identified as nurses, tend to use their contacts and short distances for the domestic situation [1, 19, 20]. They are looking for more detailed information, explanations and advice but also contacts to get better support, etc. [1, 19, 20].

### Fluidity of self-positioning (e)

Due to the fact that both roles are omnipresent, the DDCs position themselves more toward one role or the other. Furthermore, positioning itself is not static but fluid [1, 8–10, 12–14, 16, 19, 20, 23–25]. These and the associated responsibilities and activities can change suddenly at indefinite intervals and with varying intensity. In addition, the characteristics of each role, as described previously, are to be understood as occurring frequently when DDCs are positioned close to the poles; however, these characteristics can also occur when identifying with the other role. For example, DDCs who identify themselves as nurses can take over hands-on care in addition to the coordinating and organizing role, if no other relatives are available, while DDCs as family caregivers repeatedly control the care provided by the nursing staff. This example illustrates that the more a DDC is positioned towards the middle of the continuum, the higher the probability that they will show behavior that seems contrary at the same time. This behavior also depends on which contexts they are acting in: for example, in the family context they may act as a family caregiver and, at the same time, act as a professional nurse in the healthcare system context.

### Conceptual summary of all results

**Fig. 2:**
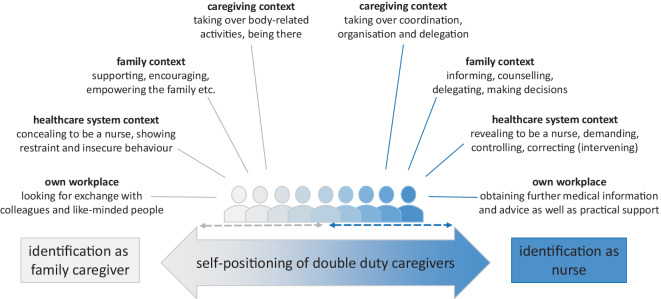
Model of the *Continuum of Self-Positioning of Double Duty Caregivers* (own illustration)

Overall, the synthesis of the results of the identified five themes leads to a conceptual model of *Continuum of Self-Positioning of Double Duty Caregivers *as shown in Fig. 2. In this respect, it should be noted that the model was developed exploratively and descriptively and has not yet been empirically tested; however, the model described here is contrasted below with a model of the phenomenon of self-positioning from a study published last year, which was developed on the basis of interviews with DDCs from Germany and Switzerland [11].

## Discussion

The results of this secondary literature review can be seen as behavior and activities which differ in both private and professional contexts, depending on whether DDCs identify more as family caregivers or as professional nurses. This shows that double duty caregiving is more complex than simply dividing caregiving responsibilities into private and professional duties. Although it seems that the described activities are related to either the family caregiver or the nurse identity, all actions can be performed in either context, with more or less intensity. This is because the self-positioning process in the private care arrangement can be understood as a continuum. Therefore, everything is fluid and not static. This also means, for example, that it is possible to console when acting as a family caregiver but also when acting as a professional nurse.

The DDCs who identify as the nurse in the care arrangement appear to have assumed a more active, confident and dominant role in the construction of medical and nursing care [1, 3, 9, 10, 12–14, 16, 19, 20, 23, 26]. Whereas DDCs in their role as family caregivers are very engaged in hands-on care they avoid taking on too much responsibility [3, 9, 13, 14, 17, 19, 20, 23, 26]. It seems that they prefer to leave the responsibility and coordination of care work to other family members or healthcare personnel. They mostly act only when there is a strong need to do so, e.g., when there exists a risk for their loved ones or there is no other person who can care for them.

Jähnke [11] also addressed the concept of DDC positioning, which corresponds with our results. She found that positioning as a family caregiver and at the same time as a nurse is a central phenomenon of DDCs. Therefore, she concluded that DDCs carry both roles inside and cannot completely abandon either. It also shows that influences, relationships, and their own ideas ensure that they would always identify themselves as a family caregiver and a nursing professional in the respective care arrangement. This forms two worlds between which the DDCs can move. All aspects of the model are tailored to the primary position as a professional nurse.

The model shown here was developed from the perspective of the identification process itself as a continuum, whereby the transition from one role to the other is fluid; however, looking at the characteristics shown by DDCs in their role as nurses, some can be assigned to the action strategies described by Jähnke [11].

The conceptual model *Continuum of Self-Positioning of Double Duty Caregivers* illustrates that the behavior and characteristics of DDCs in the role as a family caregiver differ from the role as a nurse. The DDCs as family caregivers behave more like other family caregivers and seem to have the same needs and demands. In contrast to other family caregivers, they benefit from their medical and nursing expertise, at the same time this can increase the worry about their loved one. The DDC model described in this article (a) provides the opportunity for an initial assessment of self-positioning by (b) explaining the characteristics to five different contexts, in which a DDC acts. Furthermore, the DDC model can be linked to interventions to address the needs of DDCs. Due to the fluidity of the DDC roles and positions, the next step would be to reflect, together with the DDCs, on their self-positioning within their private (as well as professional) care arrangement, to identify what kind of needs they have and what kind of support they need to fulfil their dual role.

## Limitations

As no studies from Germany could be included in the analysis for the development of the conceptual model *Continuum of Self-Positioning of Double Duty Caregivers*, it is not possible to conclusively determine whether the model is also transferable to DDCs living in Germany. Furthermore, no gender-specific statements can be made here, as even if men were present in some study samples, the characteristics described were not explicitly differentiated between men and women; however, as men are increasingly involved in both professional and informal care, there is a need for further research on this topic.

## Conclusion

Based on the two models discussed here, there is the potential to gain and develop a new and more comprehensive understanding of the complexity of double duty caregiving, especially by including findings from different perspectives. These aspects should be investigated further as they could be used for the development of other and more far-reaching action strategies, etc. if considered in greater depth and detail.

The conceptual model *Continuum of Self-Positioning of Double Duty Caregivers* describes different behaviors and characteristics shown by DDCs that can be assigned to 5 themes: caregiving contexts, family context, healthcare system context, own workplace, and fluidity of self-positioning. Knowing about and recognizing these characteristics might be helpful in determining whether DDCs positioned themselves more as a family caregiver or a nurse in the private care arrangement. This knowledge would be beneficial to all persons involved in the care arrangement and help to relieve the burden on DDCs, family members and healthcare workers.
